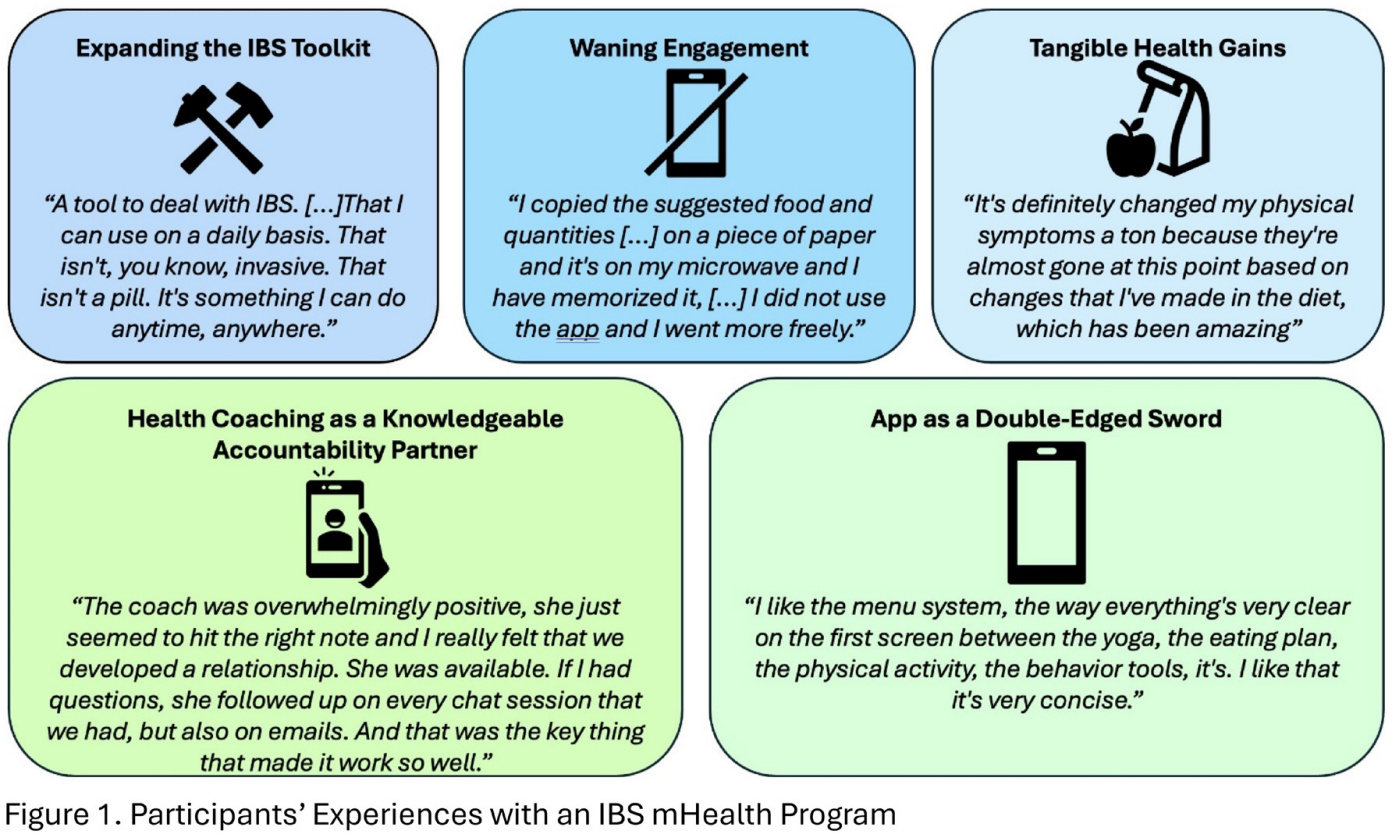# Poster Session I - A149 EMPOWEREMENT IN THE PALM OF YOUR HAND: IBS PATIENT PERSPECTIVES ON A MOBILE APP WITH HEALTH COACHING

**DOI:** 10.1093/jcag/gwaf042.149

**Published:** 2026-02-13

**Authors:** M Eisele, A D’Silva, M Yousuf, N Haskey, Y Nasser, M Raman

**Affiliations:** Internal Medicine, University of Calgary Cumming School of Medicine, Calgary, AB, Canada; Internal Medicine, University of Calgary Cumming School of Medicine, Calgary, AB, Canada; Internal Medicine, University of Calgary Cumming School of Medicine, Calgary, AB, Canada; Biology, The University of British Columbia, Vancouver, BC, Canada; Medicine, University of Calgary Cumming School of Medicine, Calgary, AB, Canada; Internal Medicine, University of Calgary Cumming School of Medicine, Calgary, AB, Canada

## Abstract

**Background:**

Irritable bowel syndrome (IBS) is a disorder of the gut-brain interaction that benefits from personalized self-management approaches combining dietary, behavioral, and psychological strategies for optimal symptom control. However, current health care systems lack the capacity and resources to deliver such comprehensive approaches. Mobile health (mHealth) interventions offer a promising avenue to fill this gap.

**Aims:**

This study explored patient experiences with LyfeMD + (LyfeMD app plus health coaching (HC)) to identify mechanisms that facilitate user engagement and to assess perceived impacts on self-management, symptom control, and overall well-being.

**Methods:**

Following a 12-week LyfeMD^+^ intervention, semi-structured interviews were analyzed using NVivo 15. LyfeMD is an app that provides evidence-based education and self-management programs on diet, physical activity, yoga-breathing-mindfulness, and behaviour change skills. HC included four remote calls and on-demand ability to communicate via the app or email. A thematic approach combining deductive coding from a predefined codebook with inductive theme development. Two coders iteratively compared and refined themes to enhance analytic rigor.

**Results:**

Interviews with 13 participants (Table 1.) revealed five overarching themes (Figure 1.): (1) Interest in expanding the IBS Management Toolkit - participants were hoping to broaden their IBS knowledge, acquire practical skills, and achieve symptom relief; (2) The app as a double-edged sword - while users valued the variety of educational content and the ease of use of the application, they also noted technical limitations, overwhelming amount of information, and lack of tracking tools; (3) HC as a knowledgeable accountability partner - coaching enhanced motivation, self-efficacy, and adherence through personalized, relational support; (4) Tangible health gains - participants described improvements in gastrointestinal symptoms and sleep, which contributed to a sense of empowerment and control over their condition; (5) Waning Engagement - use declined over time due to knowledge saturation and competing demands.

**Conclusions:**

Participants perceived LyfeMD^+^ as a useful adjunct to IBS care, valuing personalized coaching, structured education and a variety in available tools. Future enhancements should focus on improving app functionality, expanded tracking abilities, and supporting flexible engagement to optimize the user experience.

**Funding Agencies:**

None